# Crystal structures of methyl 3,5-di­methyl­benzoate, 3,5-bis­(bromo­meth­yl)phenyl acetate and 5-hy­droxy­benzene-1,3-dicarbaldehyde

**DOI:** 10.1107/S2056989022005643

**Published:** 2022-06-07

**Authors:** Ben Ebersbach, Wilhelm Seichter, Monika Mazik

**Affiliations:** a Technische Universität Bergakademie Freiberg, Leipziger Str. 29, D-09596 Freiberg/Sachsen, Germany

**Keywords:** crystal structures, 1,3,5-tris­ubstituted benzene derivatives, hydrogen bonding, C–H⋯π and π–π inter­actions

## Abstract

The crystal structures of the title compounds, methyl 3,5-di­methyl­benzoate (C_10_H_12_O_2_; **1**), 3,5-bis­(bromo­meth­yl)phenyl acetate (C_10_H_10_Br_2_O_2_; **2**) and 5-hy­droxy­benzene-1,3-dicarbaldehyde (C_8_H_6_O_3_; **3**) were determined by single-crystal X-ray analysis. The crystals of **1** are composed of strands of C—H⋯O=C bonded mol­ecules, which are further arranged into layers. As a result of the presence of two bromo­methyl substituents in compound **2**, mol­ecular dimers formed by crystallographically non-equivalent mol­ecules are connected to structurally different two-dimensional aggregates in which the bromine atoms participate in Br⋯Br bonds of type I and type II. In the case of compound **3**, which possesses three donor/acceptor substituents, the mol­ecular association in the crystal creates a close three-dimensional network comprising C_ar­yl_—H⋯O_hy­droxy_, C_form­yl_—H⋯O_form­yl_ and O—H⋯O_form­yl_ bonds.

## Abstract 2

The crystals of methyl 3,5-di­methyl­benzoate are composed of strands of C—H⋯O=C bonded mol­ecules, which are further arranged into layers. As a result of the presence of two bromo­methyl substituents in 3,5-bis­(bromo­meth­yl)phenyl acetate, mol­ecular dimers formed by crystallographically non-equivalent mol­ecules are connected to structurally different two-dimensional aggregates in which the bromine atoms participate in Br⋯Br bonds of type I and type II. In the case of 5-hy­droxy­benzene-1,3-dicarbaldehyde,which possesses three donor/acceptor substituents, the mol­ecular association in the crystal creates a close three-dimensional network comprising C_ar­yl_—H⋯O_hy­droxy_, C_form­yl_—H⋯O_form­yl_ and O—H⋯O_form­yl_ bonds.

## 1. Chemical context

Studies on mol­ecular recognition of carbohydrates by artificial receptors revealed that macrocyclic compounds bearing two flexible side-arms represent effective and selective receptors for complexation of gluco­pyran­osides. The binding properties of these compounds depend on the nature of their building blocks, among others, the type of bridging units that connect two aromatic platforms (Lippe & Mazik, 2013 ▸, 2015 ▸; Amrhein *et al.*, 2016 ▸, 2021 ▸; Amrhein & Mazik, 2021 ▸). The design of such receptor architectures was inspired by the results of our crystallographic studies on receptor–carbohydrate complexes (Mazik *et al.*, 2005 ▸; for recent examples, see Köhler *et al.*, 2020 ▸, 2021 ▸). For the syntheses of macrocycles consisting of benzene-based bridges, various 2- or 5-substituted benzene-1,3-di­carb­aldehydes have proven to be useful starting materials. Benzene derivatives with methyl or bromo­methyl groups in positions 1 and 3 are used to prepare the latter compounds. The crystal structures of three 1,3,5-substituted benzenes, serving as precursors for the syntheses of the macrocyclic compounds mentioned above, are described in this work. 
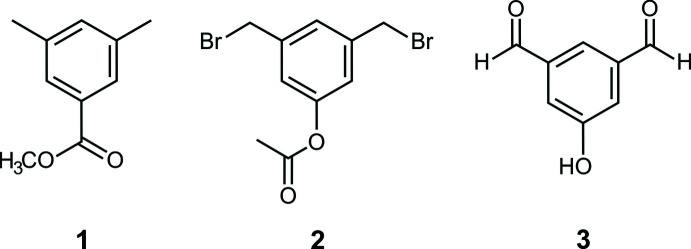


## 2. Structural commentary

**Figure 1:**
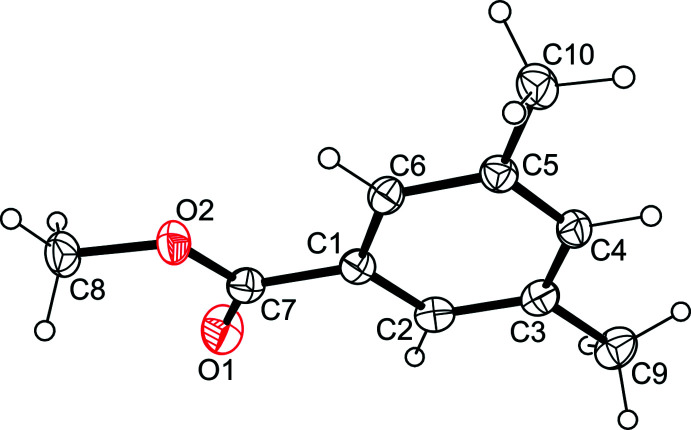
Perspective view of the mol­ecular structure of **1**. Anisotropic displacement ellipsoids are drawn at the 50% probability level.

**Figure 2:**
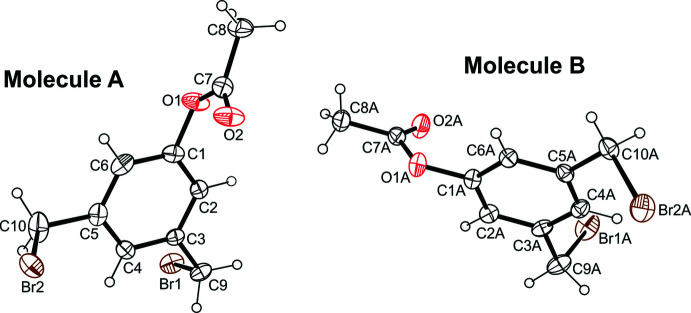
Perspective view of the mol­ecular structure of **2**. Anisotropic displacement ellipsoids are drawn at the 50% probability level.

**Figure 3:**
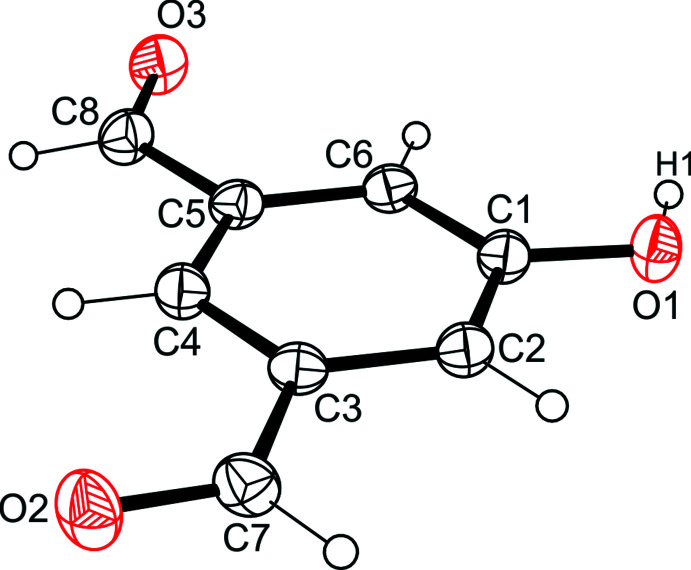
Perspective view of the mol­ecular structure of **3**. Anisotropic displacement ellipsoids are drawn at the 50% probability level.

The title compounds **1** and **3** crystallize in the monoclinic system (space group *P*2_1_/*c*, *Z* = 4), whereas compound **2** crystallizes in the triclinic space group *P* 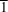 with two independent but conformationally similar mol­ecules (*A* and *B*) in the asymmetric unit of the cell. In compound **1** (Fig. 1 ▸), the plane through the methyl­oxycarbonyl unit is tilted at an angle of 8.70 (8) ° with respect to the benzene ring. In the independent mol­ecules of **2** (Fig. 2 ▸), the planes passing through the ester units are inclined at angles of 62.9 (1) and 81.3 (1)°, respectively, to the plane of their arene ring. The two bromine atoms of each mol­ecule are located on opposite sides of the benzene ring. In the crystal of the 5-hy­droxy­benzene-1,3-dicarbaldehyde (**3**) (Fig. 3 ▸), the mol­ecule deviates slightly from planarity, with the formyl groups rotated out of the benzene ring at angles of 4.43 (16) and 4.04 (16)°.

## 3. Supra­molecular features

**Figure 4:**
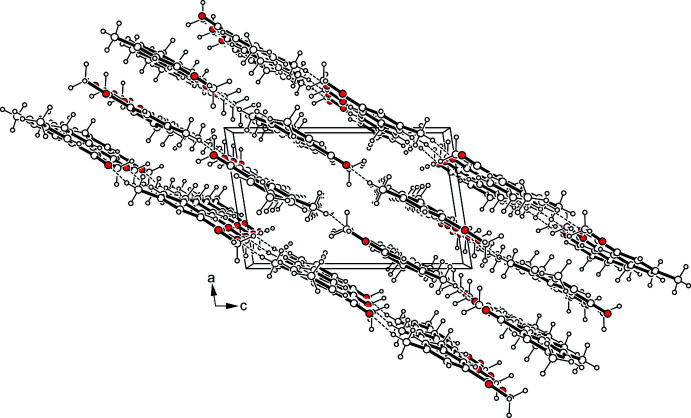
Packing diagram of **1** viewed down the crystallographic *b*-axis.

**Figure 5:**
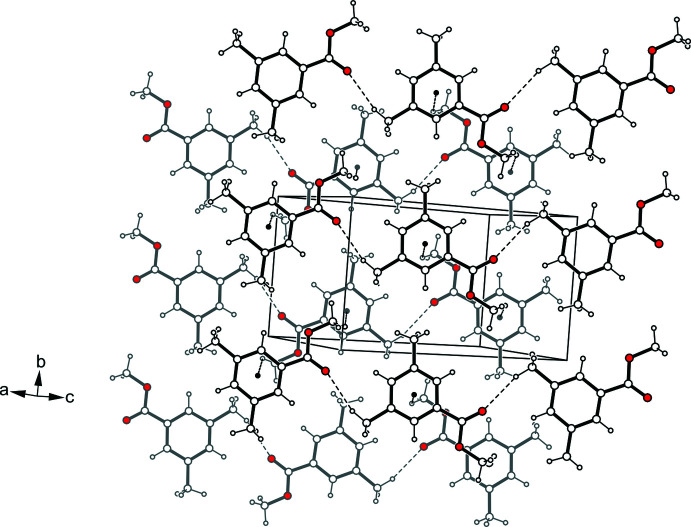
Excerpt of the packing structure of **1** viewed in the direction of the layer normal. Dashed lines represent hydrogen-bonding inter­actions.

In the crystal structure of **1**, the mol­ecules are arranged into layers extending parallel to the crystallographic [101] plane (see Fig. 4 ▸). Within a given layer, the mol­ecules are linked in strands *via* C—H⋯O=C bonds [*d*(H⋯O) 2.57 Å; Table 1 ▸], with a methyl H atom acting as the donor. No directional inter­actions are present between the mol­ecular strands of a layer. With the participation of a H atom of the methyl ester unit, the linkage between the mol­ecules of adjacent layers occurs by C—H⋯π contacts (Nishio *et al.*, 2009 ▸) with a H⋯*Cg* distance of 2.77 Å. Fig. 5 ▸ shows a packing excerpt of the crystal structure viewed in the direction of the layer normal.

**Figure 6:**
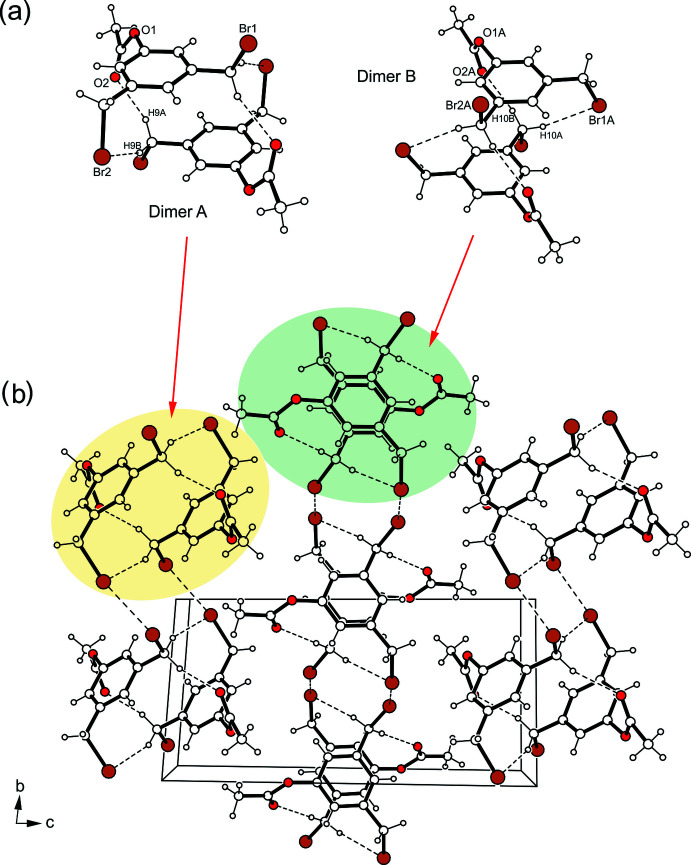
(*a*) Structures of the dimers formed by mol­ecule *A* (left) and mol­ecule *B* (right) in the crystal structure of **2**. (*b*) Packing structure of **2** viewed down the *a*-axis. Hydrogen bonds and Br⋯Br inter­actions are shown as dashed lines.

**Figure 7:**
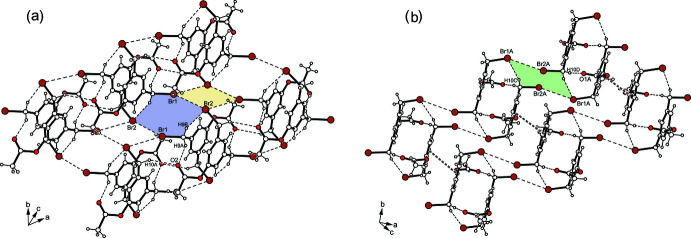
Patterns of inter­molecular inter­actions created by (*a*) mol­ecule *A* and (*b*) mol­ecule *B* in the crystal structure of **2**.

The excerpt of the crystal structure of **2** shown in Fig. 6 ▸ reveals two different inversion-symmetric dimers as the smallest supra­molecular entities, in which the mol­ecules are linked in an identical manner by C—H⋯O=C and C—H⋯Br bonds (Table 2 ▸) (Desiraju & Steiner, 1999 ▸). These dimers, however, form differently structured domains within the crystal. The dimers formed by mol­ecule *A* are connected *via* Br⋯Br bonds (Pedireddy *et al.*, 1999 ▸) of type I [*d*(Br⋯Br) = 3.562 (1) Å; θ_1_ = 150.2°, θ_2_ = 158.5°] and of type II [*d*(Br⋯Br) = 3.859 (1) Å; θ_1_ = 135.0°, θ_2_ = 84.6°] as well as C—H⋯Br hydrogen bonds to form two-dimensional aggregates extending parallel to crystallographic [011] plane, in which the bromine atoms contribute to the formation of a cyclic four-membered synthon (Br_4_) and an eight-membered bonding motif (Fig. 7 ▸ *a*). The structure of the domains created by mol­ecule *B* is fundamentally different from those formed by mol­ecule *A*. In them, the dimers are linked in a strand-like fashion *via* type I Br⋯Br inter­actions [*d*(Br⋯Br) = 3.638 (1) Å; *θ*_1_ = 152.3°, θ_2_ = 145.9°] (Fig. 7 ▸ *b*), which are part of an eight-membered ring motif. In the direction of the crystallographic *a*-axis, the connection of the dimers occurs through π–·π (face-to-face) inter­actions (Tiekink & Zukerman-Schpector, 2012 ▸) with a centroid–centroid distance of 3.653 (1) Å and an offset of 1.592 Å between the inter­acting arene rings.

**Figure 8:**
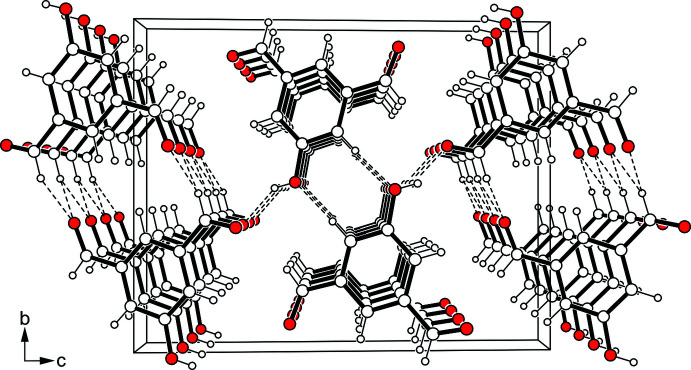
Packing diagram of **3** viewed down the *a*-axis. Dashed lines represent hydrogen bonds.

**Figure 9:**
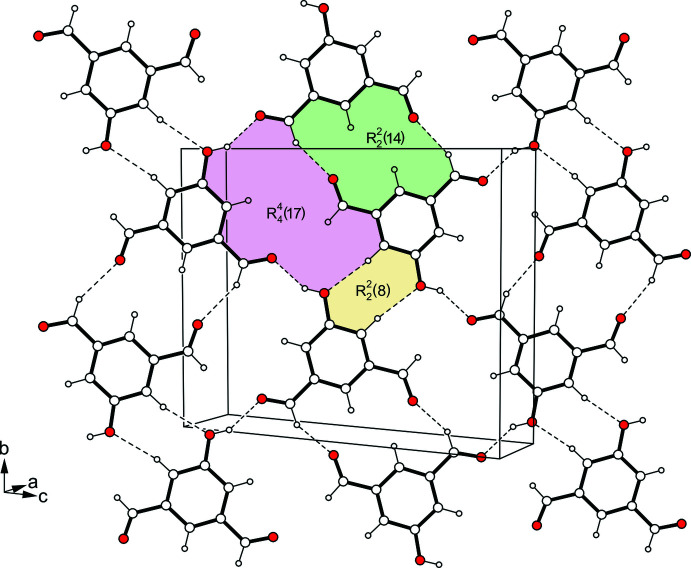
Mode of inter­molecular non-covalent inter­actions in the crystal structure of **3**. The cyclic supra­molecular synthons are marked by colour highlighting.

Viewing the crystal structure of compound **3** in the direction of the *a*-axis reveals a stacking arrangement of mol­ecules (Fig. 8 ▸). Along the stacking axis the centroid-centroid distance of 3.735 (1) Å between consecutive mol­ecules indicates the presence of offset π–π inter­actions. As is obvious from Fig. 9 ▸, showing the mode of non-covalent bonding in the crystal, the H atom of the hy­droxy group forms an inter­molecular O—H⋯O bond [O1—H1⋯O3 = 1.91 (2) Å, 150 (2)°; Table 3 ▸], while its O atom forms a C—H⋯O bond [C2—H2⋯O1 = 2.43 Å, 159.6°; Table 3 ▸], thus creating a supra­molecular synthon with the graph set 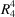 (17) (Etter, 1990 ▸; Etter *et al.*, 1990 ▸; Bernstein *et al.*, 1995 ▸) in which four mol­ecules take part. The OH group is also involved in formation of an inversion-symmetric ring motif of the structure 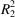 (8). Another supra­molecular motif corresponding to the 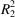 (14) graph set is formed by the formyl groups of inversion-related mol­ecules.

## 4. Database survey

A search in the Cambridge Structural Database (CSD, Version 5.43, update November 2021; Groom *et al.*, 2016 ▸) for benzene derivates containing the corresponding substituents resulted in several hits, but with relatively strong structural differences from the searched structures. The compound with the closest relation to **1** is ethyl 2,3,5,6-tetra­methyl­benzoate (FICVET; Pinkus *et al.* 2005 ▸), the crystal structure of which features C—H⋯O and C—H⋯π inter­actions. In the case of bromo­methyl-substituted benzenes, the crystal structures of 1,2,4,5-tetra­kis­(bromo­meth­yl)-3,6-di­meth­oxy­benzene, 1,2,4,5-tetra­kis­(bromo­meth­yl)-3,6-bis­(hex­yloxy)benzene and 1,2,4,5-tetra­­kis­(bromo­meth­yl)-3,6-bis­(2-ethyl­but­oxy)benzene (BAS­ZIG, BASZOM, BASZUS; Velde *et al.* 2012 ▸) as well as 1,3,5-tris­(bromo­meth­yl)-2,4,6-tri­meth­oxy­benzene (IDOBAG; Koch *et al.* 2013 ▸) are worth mentioning. The crystal structure of IDOBAG, for example, is characterized by the presence of C—H⋯O and C—H⋯Br hydrogen bonds as well as C—Br⋯Br halogen bonds of type II, as observed also in the crystal structure of **2**. In the crystal structure of 2-hy­droxy­isophthalaldehyde (NEJJOB; Zondervan *et al.* 1997 ▸), an analogue of **3**, the mol­ecules inter­act *via* O—H⋯O hydrogen bonds, forming chains. In addition, the hy­droxy group is involved in an intra­molecular O—H⋯O hydrogen bond with the neighbouring carbonyl oxygen atom.

## 5. Synthesis and crystallization

Compounds **1**–**3** were prepared according to literature procedures (Kurz & Göbel, 1996 ▸; Battaini *et al.*, 2003 ▸; Star *et al.*, 2003 ▸).

Suitable crystals of compounds **2** and **3** for X-ray analysis were obtained by slow evaporation from a hexane solution, while crystals of **1** were grown from a subcooled melt.

## 6. Refinement

Crystal data, data collection and structure refinement details are summarized in Table 4 ▸. Hydrogen atom H1 in **3** was located in a difference-Fourier map and freely refined. Other H atoms were positioned geometrically and refined isotropically using a riding model with C—H = 0.93–0.98 Å and *U*_iso_(H) = 1.2–1.5*U*_eq_(C).

## Supplementary Material

Crystal structure: contains datablock(s) 1, 2, 3, global. DOI: 10.1107/S2056989022005643/ex2057sup1.cif

Structure factors: contains datablock(s) 1. DOI: 10.1107/S2056989022005643/ex20571sup4.hkl

Supporting information file. DOI: 10.1107/S2056989022005643/ex20571sup5.cml

Structure factors: contains datablock(s) 2. DOI: 10.1107/S2056989022005643/ex20572sup2.hkl

Supporting information file. DOI: 10.1107/S2056989022005643/ex20572sup6.cml

Structure factors: contains datablock(s) 3. DOI: 10.1107/S2056989022005643/ex20573sup3.hkl

Supporting information file. DOI: 10.1107/S2056989022005643/ex20573sup7.cml

CCDC references: 2174617, 2174616, 2174615

Additional supporting information:  crystallographic information; 3D view; checkCIF report

## Figures and Tables

**Table 1:** Hydrogen-bond geometry (Å, °) for **1** [Chem scheme1] *Cg*1 represents the centroid of the C1–C6 ring.

*D*—H⋯*A*	*D*—H	H⋯*A*	*D*⋯*A*	*D*—H⋯*A*
C10—H10*B*⋯O1^i^	0.98	2.57	3.5215 (19)	163
C8—H8*B*⋯*Cg*1^ii^	0.98	2.76	3.445 (2)	127

Symmetry codes: (i) 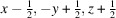 ; (ii) 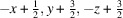 .

**Table 2:** Hydrogen-bond geometry (Å, °) for **2** [Chem scheme1]

*D*—H⋯*A*	*D*—H	H⋯*A*	*D*⋯*A*	*D*—H⋯*A*
C10*A*—H10*D*⋯O2*A* ^i^	0.97	2.28	3.236 (3)	168
C10*A*—H10*C*⋯Br1*A* ^i^	0.97	2.89	3.836 (3)	164
C8*A*—H8*A*3⋯O2	0.96	2.58	3.521 (4)	168
C10—H10*B*⋯Br2*A* ^i^	0.97	3.01	3.757 (3)	135
C10—H10*A*⋯O2^ii^	0.97	2.58	3.449 (3)	150
C9—H9*B*⋯Br2^iii^	0.97	2.95	3.854 (3)	156
C9—H9*A*⋯O2^iii^	0.97	2.45	3.334 (3)	151

Symmetry codes: (i) 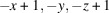 ; (ii) 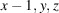 ; (iii) 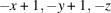 .

**Table 3:** Hydrogen-bond geometry (Å, °) for **3** [Chem scheme1]

*D*—H⋯*A*	*D*—H	H⋯*A*	*D*⋯*A*	*D*—H⋯*A*
C2—H2⋯O1^i^	0.95	2.43	3.3354 (16)	160
C8—H8⋯O2^ii^	0.95	2.58	3.1973 (18)	123
O1—H1⋯O3^iii^	0.85 (2)	1.91 (2)	2.6795 (13)	150 (2)

Symmetry codes: (i) 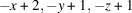 ; (ii) 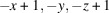 ; (iii) 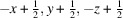 .

**Table 4:** Experimental details

	**1**	**2**	**3**
Crystal data			
Chemical formula	C_10_H_12_O_2_	C_10_H_10_Br_2_O_2_	C_8_H_6_O_3_
*M*_r_	164.20	322.00	150.13
Crystal system, space group	Monoclinic, *P*2_1_/*n*	Triclinic, *P* 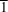	Monoclinic, *P*2_1_/*n*
Temperature (K)	153	130	153
*a*, *b*, *c* (Å)	8.4631 (6), 7.9793 (4), 13.4042 (9)	7.7936 (2), 9.1655 (2), 17.2292 (4)	3.7345 (1), 11.9549 (4), 15.0846 (5)
α, β, γ (°)	90, 98.835 (6), 90	88.1637 (12), 80.9050 (12), 65.8659 (11)	90, 94.212 (2), 90
*V* (Å^3^)	894.44 (10)	1108.30 (5)	671.64 (4)
*Z*	4	4	4
Radiation type	Mo *K*α	Mo *K*α	Mo *K*α
μ (mm^−1^)	0.08	7.29	0.12
Crystal size (mm)	0.40 × 0.25 × 0.16	0.46 × 0.39 × 0.27	0.42 × 0.28 × 0.19

Data collection			
Diffractometer	Stoe IPDS 2T	Bruker Kappa APEXII CCD area detector	Bruker Kappa APEXII CCD area detector
Absorption correction	–	Multi-scan (*SADABS*; Bruker, 2014 ▸)	–
*T*_min_, *T*_max_	–	0.134, 0.244	–
No. of measured, independent and observed [*I* > 2σ(*I*)] reflections	7437, 1762, 1449	29065, 5842, 5305	11533, 1819, 1519
*R*_int_	0.046	0.033	0.058
(sin θ/λ)_max_ (Å^−1^)	0.617	0.680	0.691

Refinement			
*R*[*F* ^2^ > 2σ(*F* ^2^)], *wR*(*F* ^2^), *S*	0.041, 0.116, 1.05	0.028, 0.070, 1.04	0.047, 0.131, 1.06
No. of reflections	1762	5842	1819
No. of parameters	112	255	104
H-atom treatment	H-atom parameters constrained	H-atom parameters constrained	H atoms treated by a mixture of independent and constrained refinement
Δρ_max_, Δρ_min_ (e Å^−3^)	0.24, −0.19	1.21, −0.98	0.33, −0.28

Computer programs: *X-AREA* and *X-RED* (Stoe & Cie, 2002 ▸), *APEX2* and *SAINT* (Bruker, 2014 ▸), *SIR2014* (Burla *et al.*, 2015 ▸), *SHELXS97* (Sheldrick, 2008 ▸), *SHELXL* (Sheldrick, 2015 ▸), *ShelXle* (Hübschle *et al.*, 2011 ▸), *XP* (Sheldrick, 2008 ▸), *ORTEP-3 for Windows* and *WinGX* (Farrugia, 2012 ▸) and *publCIF* (Westrip, 2010 ▸).

## supplementary crystallographic information

### Methyl 3,5-dimethylbenzoate (1) Crystal data

**Table:**

C_10_H_12_O_2_	*F*(000) = 352
*M**_r_* = 164.20	*D*_x_ = 1.219 Mg m^−^^3^
Monoclinic, *P*2_1_/*n*	Mo *K*α radiation, λ = 0.71073 Å
*a* = 8.4631 (6) Å	Cell parameters from 7437 reflections
*b* = 7.9793 (4) Å	θ = 2.7–27.2°
*c* = 13.4042 (9) Å	µ = 0.08 mm^−^^1^
β = 98.835 (6)°	*T* = 153 K
*V* = 894.44 (10) Å^3^	Piece, colorless
*Z* = 4	0.40 × 0.25 × 0.16 mm

### Methyl 3,5-dimethylbenzoate (1) Data collection

**Table:**

Stoe IPDS 2T diffractometer	1449 reflections with *I* > 2σ(*I*)
Radiation source: sealed X-ray tube, 12 x 0.4 mm long-fine focus	*R*_int_ = 0.046
Plane graphite monochromator	θ_max_ = 26.0°, θ_min_ = 2.7°
Detector resolution: 6.67 pixels mm^-1^	*h* = −10→9
rotation method scans	*k* = −9→9
7437 measured reflections	*l* = −16→16
1762 independent reflections	

### Methyl 3,5-dimethylbenzoate (1) Refinement

**Table:**

Refinement on *F*^2^	0 restraints
Least-squares matrix: full	Hydrogen site location: inferred from neighbouring sites
*R*[*F*^2^ > 2σ(*F*^2^)] = 0.041	H-atom parameters constrained
*wR*(*F*^2^) = 0.116	*w* = 1/[σ^2^(*F*_o_^2^) + (0.0548*P*)^2^ + 0.2723*P*] where *P* = (*F*_o_^2^ + 2*F*_c_^2^)/3
*S* = 1.05	(Δ/σ)_max_ < 0.001
1762 reflections	Δρ_max_ = 0.24 e Å^−^^3^
112 parameters	Δρ_min_ = −0.19 e Å^−^^3^

### Methyl 3,5-dimethylbenzoate (1) Special details

**Table:**

Geometry. All esds (except the esd in the dihedral angle between two l.s. planes) are estimated using the full covariance matrix. The cell esds are taken into account individually in the estimation of esds in distances, angles and torsion angles; correlations between esds in cell parameters are only used when they are defined by crystal symmetry. An approximate (isotropic) treatment of cell esds is used for estimating esds involving l.s. planes.

### Methyl 3,5-dimethylbenzoate (1) Fractional atomic coordinates and isotropic or equivalent isotropic displacement parameters (Å^2^)

**Table:**

	*x*	*y*	*z*	*U*_iso_*/*U*_eq_	
O1	0.27708 (14)	0.33954 (14)	0.48553 (8)	0.0433 (3)	
O2	0.20755 (12)	0.58613 (12)	0.54594 (7)	0.0309 (3)	
C1	0.12649 (14)	0.34326 (16)	0.62305 (9)	0.0244 (3)	
C2	0.10405 (16)	0.16993 (17)	0.62357 (10)	0.0276 (3)	
H2	0.1406	0.1027	0.5733	0.033*	
C3	0.02860 (16)	0.09507 (16)	0.69720 (10)	0.0283 (3)	
C4	−0.02303 (16)	0.19629 (17)	0.77063 (10)	0.0279 (3)	
H4	−0.0747	0.1458	0.8211	0.033*	
C5	−0.00096 (15)	0.36934 (17)	0.77210 (10)	0.0257 (3)	
C6	0.07405 (15)	0.44202 (17)	0.69705 (10)	0.0249 (3)	
H6	0.0894	0.5599	0.6965	0.030*	
C7	0.21123 (15)	0.41859 (17)	0.54403 (10)	0.0271 (3)	
C8	0.29088 (18)	0.6711 (2)	0.47431 (11)	0.0361 (4)	
H8A	0.2808	0.7926	0.4822	0.054*	
H8B	0.2442	0.6387	0.4056	0.054*	
H8C	0.4042	0.6398	0.4866	0.054*	
C9	0.00480 (19)	−0.09303 (17)	0.69749 (12)	0.0383 (4)	
H9A	−0.0136	−0.1291	0.7647	0.057*	
H9B	0.1005	−0.1487	0.6805	0.057*	
H9C	−0.0879	−0.1231	0.6475	0.057*	
C10	−0.05669 (18)	0.47866 (18)	0.85204 (11)	0.0333 (3)	
H10A	0.0340	0.5428	0.8870	0.050*	
H10B	−0.1010	0.4080	0.9008	0.050*	
H10C	−0.1392	0.5560	0.8201	0.050*	

### Methyl 3,5-dimethylbenzoate (1) Atomic displacement parameters (Å^2^)

**Table:**

	*U*^11^	*U*^22^	*U*^33^	*U*^12^	*U*^13^	*U*^23^
O1	0.0488 (7)	0.0417 (6)	0.0461 (7)	0.0006 (5)	0.0280 (5)	−0.0072 (5)
O2	0.0345 (6)	0.0299 (6)	0.0308 (5)	−0.0021 (4)	0.0126 (4)	0.0040 (4)
C1	0.0207 (6)	0.0273 (7)	0.0253 (7)	0.0015 (5)	0.0035 (5)	−0.0001 (5)
C2	0.0262 (7)	0.0260 (7)	0.0300 (7)	0.0039 (5)	0.0025 (5)	−0.0042 (5)
C3	0.0269 (7)	0.0232 (7)	0.0329 (7)	0.0006 (5)	−0.0012 (5)	0.0017 (5)
C4	0.0283 (7)	0.0284 (7)	0.0265 (7)	−0.0026 (5)	0.0023 (5)	0.0046 (5)
C5	0.0249 (7)	0.0274 (7)	0.0247 (6)	0.0005 (5)	0.0034 (5)	−0.0001 (5)
C6	0.0245 (6)	0.0221 (6)	0.0280 (7)	0.0004 (5)	0.0040 (5)	0.0004 (5)
C7	0.0223 (6)	0.0316 (7)	0.0276 (7)	0.0001 (5)	0.0045 (5)	−0.0024 (5)
C8	0.0320 (8)	0.0441 (9)	0.0337 (8)	−0.0061 (6)	0.0098 (6)	0.0092 (6)
C9	0.0418 (9)	0.0237 (8)	0.0480 (9)	−0.0012 (6)	0.0024 (7)	0.0011 (6)
C10	0.0388 (8)	0.0339 (8)	0.0300 (7)	−0.0009 (6)	0.0140 (6)	−0.0028 (6)

### Methyl 3,5-dimethylbenzoate (1) Geometric parameters (Å, º)

**Table:**

O1—C7	1.2073 (16)	C5—C6	1.3956 (18)
O2—C7	1.3375 (17)	C5—C10	1.5121 (18)
O2—C8	1.4448 (16)	C6—H6	0.9500
C1—C6	1.3917 (18)	C8—H8A	0.9800
C1—C2	1.3961 (19)	C8—H8B	0.9800
C1—C7	1.4936 (17)	C8—H8C	0.9800
C2—C3	1.3900 (19)	C9—H9A	0.9800
C2—H2	0.9500	C9—H9B	0.9800
C3—C4	1.3944 (19)	C9—H9C	0.9800
C3—C9	1.5144 (19)	C10—H10A	0.9800
C4—C5	1.3931 (19)	C10—H10B	0.9800
C4—H4	0.9500	C10—H10C	0.9800
			
C7—O2—C8	116.20 (11)	O1—C7—C1	124.76 (13)
C6—C1—C2	119.92 (12)	O2—C7—C1	111.94 (11)
C6—C1—C7	121.19 (12)	O2—C8—H8A	109.5
C2—C1—C7	118.86 (12)	O2—C8—H8B	109.5
C3—C2—C1	120.46 (12)	H8A—C8—H8B	109.5
C3—C2—H2	119.8	O2—C8—H8C	109.5
C1—C2—H2	119.8	H8A—C8—H8C	109.5
C2—C3—C4	118.70 (12)	H8B—C8—H8C	109.5
C2—C3—C9	120.23 (13)	C3—C9—H9A	109.5
C4—C3—C9	121.07 (13)	C3—C9—H9B	109.5
C5—C4—C3	121.89 (12)	H9A—C9—H9B	109.5
C5—C4—H4	119.1	C3—C9—H9C	109.5
C3—C4—H4	119.1	H9A—C9—H9C	109.5
C4—C5—C6	118.46 (12)	H9B—C9—H9C	109.5
C4—C5—C10	121.74 (12)	C5—C10—H10A	109.5
C6—C5—C10	119.80 (12)	C5—C10—H10B	109.5
C1—C6—C5	120.57 (12)	H10A—C10—H10B	109.5
C1—C6—H6	119.7	C5—C10—H10C	109.5
C5—C6—H6	119.7	H10A—C10—H10C	109.5
O1—C7—O2	123.30 (12)	H10B—C10—H10C	109.5
			
C6—C1—C2—C3	0.43 (19)	C7—C1—C6—C5	−178.18 (12)
C7—C1—C2—C3	178.69 (11)	C4—C5—C6—C1	−0.48 (19)
C1—C2—C3—C4	−0.42 (19)	C10—C5—C6—C1	179.82 (12)
C1—C2—C3—C9	−179.91 (13)	C8—O2—C7—O1	−1.2 (2)
C2—C3—C4—C5	0.0 (2)	C8—O2—C7—C1	178.09 (11)
C9—C3—C4—C5	179.44 (13)	C6—C1—C7—O1	170.41 (14)
C3—C4—C5—C6	0.5 (2)	C2—C1—C7—O1	−7.8 (2)
C3—C4—C5—C10	−179.82 (12)	C6—C1—C7—O2	−8.91 (17)
C2—C1—C6—C5	0.03 (19)	C2—C1—C7—O2	172.85 (12)

### Methyl 3,5-dimethylbenzoate (1) Hydrogen-bond geometry (Å, º)

*Cg*1 represents the centroid of the C1–C6 ring.

**Table:**

*D*—H···*A*	*D*—H	H···*A*	*D*···*A*	*D*—H···*A*
C10—H10*B*···O1^i^	0.98	2.57	3.5215 (19)	163
C8—H8*B*···*Cg*1^ii^	0.98	2.76	3.445 (2)	127

Symmetry codes: (i) *x*−1/2, −*y*+1/2, *z*+1/2; (ii) −*x*+1/2, *y*+3/2, −*z*+3/2.

### 3,5-Bis(bromomethyl)phenyl acetate (2) Crystal data

**Table:**

C_10_H_10_Br_2_O_2_	*Z* = 4
*M**_r_* = 322.00	*F*(000) = 624
Triclinic, *P*1	*D*_x_ = 1.930 Mg m^−^^3^
*a* = 7.7936 (2) Å	Mo *K*α radiation, λ = 0.71073 Å
*b* = 9.1655 (2) Å	Cell parameters from 9654 reflections
*c* = 17.2292 (4) Å	θ = 2.7–36.8°
α = 88.1637 (12)°	µ = 7.29 mm^−^^1^
β = 80.9050 (12)°	*T* = 130 K
γ = 65.8659 (11)°	Irregular, colourless
*V* = 1108.30 (5) Å^3^	0.46 × 0.39 × 0.27 mm

### 3,5-Bis(bromomethyl)phenyl acetate (2) Data collection

**Table:**

Bruker Kappa APEXII CCD area detector diffractometer	5305 reflections with *I* > 2σ(*I*)
φ and ω scans	*R*_int_ = 0.033
Absorption correction: multi-scan (SADABS; Bruker, 2014)	θ_max_ = 28.9°, θ_min_ = 1.2°
*T*_min_ = 0.134, *T*_max_ = 0.244	*h* = −10→10
29065 measured reflections	*k* = −12→12
5842 independent reflections	*l* = −23→22

### 3,5-Bis(bromomethyl)phenyl acetate (2) Refinement

**Table:**

Refinement on *F*^2^	0 restraints
Least-squares matrix: full	Hydrogen site location: inferred from neighbouring sites
*R*[*F*^2^ > 2σ(*F*^2^)] = 0.028	H-atom parameters constrained
*wR*(*F*^2^) = 0.070	*w* = 1/[σ^2^(*F*_o_^2^) + (0.0273*P*)^2^ + 2.052*P*] where *P* = (*F*_o_^2^ + 2*F*_c_^2^)/3
*S* = 1.04	(Δ/σ)_max_ = 0.001
5842 reflections	Δρ_max_ = 1.21 e Å^−^^3^
255 parameters	Δρ_min_ = −0.98 e Å^−^^3^

### 3,5-Bis(bromomethyl)phenyl acetate (2) Special details

**Table:**

Geometry. All esds (except the esd in the dihedral angle between two l.s. planes) are estimated using the full covariance matrix. The cell esds are taken into account individually in the estimation of esds in distances, angles and torsion angles; correlations between esds in cell parameters are only used when they are defined by crystal symmetry. An approximate (isotropic) treatment of cell esds is used for estimating esds involving l.s. planes.

### 3,5-Bis(bromomethyl)phenyl acetate (2) Fractional atomic coordinates and isotropic or equivalent isotropic displacement parameters (Å^2^)

**Table:**

	*x*	*y*	*z*	*U*_iso_*/*U*_eq_	
Br1	−0.08475 (4)	0.81672 (3)	0.00562 (2)	0.02904 (7)	
Br2	0.48472 (4)	0.08778 (3)	0.11939 (2)	0.03302 (8)	
O1	0.5485 (3)	0.6991 (2)	0.19580 (10)	0.0223 (3)	
O2	0.8281 (3)	0.4838 (2)	0.16971 (12)	0.0300 (4)	
C1	0.4550 (3)	0.6259 (3)	0.15806 (14)	0.0180 (4)	
C2	0.3764 (3)	0.7009 (3)	0.09361 (13)	0.0177 (4)	
H2	0.3906	0.7927	0.0756	0.021*	
C3	0.2757 (3)	0.6375 (3)	0.05580 (13)	0.0168 (4)	
C4	0.2561 (3)	0.5002 (3)	0.08395 (14)	0.0183 (4)	
H4	0.1896	0.4570	0.0587	0.022*	
C5	0.3346 (3)	0.4268 (3)	0.14936 (14)	0.0189 (4)	
C6	0.4351 (3)	0.4903 (3)	0.18721 (14)	0.0190 (4)	
H6	0.4879	0.4425	0.2312	0.023*	
C7	0.7388 (4)	0.6174 (3)	0.19617 (14)	0.0204 (4)	
C8	0.8159 (4)	0.7190 (3)	0.23218 (15)	0.0262 (5)	
H8A	0.9257	0.6518	0.2548	0.039*	
H8B	0.7203	0.7887	0.2725	0.039*	
H8C	0.8515	0.7817	0.1925	0.039*	
C9	0.1944 (3)	0.7141 (3)	−0.01535 (14)	0.0221 (5)	
H9A	0.2349	0.6337	−0.0575	0.026*	
H9B	0.2432	0.7937	−0.0326	0.026*	
C10	0.3063 (4)	0.2828 (3)	0.18047 (17)	0.0258 (5)	
H10A	0.1767	0.2972	0.1783	0.031*	
H10B	0.3249	0.2712	0.2351	0.031*	
Br1A	0.43346 (3)	0.44424 (3)	0.61006 (2)	0.02337 (6)	
Br2A	0.92345 (4)	−0.40729 (3)	0.60976 (2)	0.02744 (7)	
O1A	0.9262 (3)	0.0059 (2)	0.34359 (11)	0.0285 (4)	
O2A	0.6523 (3)	0.1522 (3)	0.30204 (12)	0.0443 (6)	
C1A	0.8337 (3)	0.0113 (3)	0.42092 (14)	0.0200 (4)	
C2A	0.7921 (3)	0.1409 (3)	0.47025 (16)	0.0219 (5)	
H2A	0.8118	0.2296	0.4509	0.026*	
C3A	0.7203 (3)	0.1375 (3)	0.54912 (15)	0.0210 (5)	
C4A	0.6907 (3)	0.0042 (3)	0.57655 (14)	0.0192 (4)	
H4A	0.6413	0.0022	0.6292	0.023*	
C5A	0.7340 (3)	−0.1266 (3)	0.52649 (13)	0.0171 (4)	
C6A	0.8055 (3)	−0.1220 (3)	0.44763 (13)	0.0178 (4)	
H6A	0.8340	−0.2079	0.4133	0.021*	
C7A	0.8205 (4)	0.0866 (3)	0.28849 (14)	0.0219 (5)	
C8A	0.9420 (4)	0.0791 (4)	0.21112 (16)	0.0317 (6)	
H8A1	0.9001	0.0369	0.1712	0.048*	
H8A2	1.0722	0.0109	0.2145	0.048*	
H8A3	0.9317	0.1846	0.1980	0.048*	
C9A	0.6879 (4)	0.2709 (3)	0.60537 (19)	0.0323 (6)	
H9A1	0.7829	0.3134	0.5893	0.039*	
H9A2	0.7036	0.2291	0.6574	0.039*	
C10A	0.7074 (3)	−0.2710 (3)	0.55685 (15)	0.0225 (5)	
H10C	0.6986	−0.3315	0.5136	0.027*	
H10D	0.5896	−0.2386	0.5938	0.027*	

### 3,5-Bis(bromomethyl)phenyl acetate (2) Atomic displacement parameters (Å^2^)

**Table:**

	*U*^11^	*U*^22^	*U*^33^	*U*^12^	*U*^13^	*U*^23^
Br1	0.02417 (13)	0.02849 (13)	0.03098 (14)	−0.00553 (10)	−0.01021 (10)	0.00568 (10)
Br2	0.03039 (14)	0.01534 (12)	0.05357 (18)	−0.00887 (10)	−0.00889 (12)	0.00249 (11)
O1	0.0273 (9)	0.0173 (8)	0.0254 (9)	−0.0095 (7)	−0.0116 (7)	0.0010 (7)
O2	0.0238 (9)	0.0277 (10)	0.0385 (11)	−0.0103 (8)	−0.0035 (8)	−0.0084 (8)
C1	0.0183 (10)	0.0160 (10)	0.0198 (11)	−0.0063 (8)	−0.0051 (8)	−0.0011 (8)
C2	0.0205 (10)	0.0141 (10)	0.0182 (10)	−0.0069 (8)	−0.0028 (8)	0.0009 (8)
C3	0.0170 (10)	0.0163 (10)	0.0139 (10)	−0.0042 (8)	−0.0005 (8)	−0.0014 (8)
C4	0.0159 (10)	0.0169 (10)	0.0218 (11)	−0.0068 (8)	−0.0020 (8)	−0.0019 (8)
C5	0.0152 (10)	0.0162 (10)	0.0228 (11)	−0.0055 (8)	0.0010 (8)	0.0012 (8)
C6	0.0194 (10)	0.0174 (10)	0.0186 (10)	−0.0056 (8)	−0.0049 (8)	0.0037 (8)
C7	0.0248 (11)	0.0231 (11)	0.0166 (10)	−0.0126 (9)	−0.0045 (9)	0.0028 (9)
C8	0.0337 (13)	0.0302 (13)	0.0228 (12)	−0.0199 (11)	−0.0081 (10)	0.0016 (10)
C9	0.0238 (11)	0.0252 (12)	0.0163 (11)	−0.0088 (9)	−0.0040 (9)	0.0007 (9)
C10	0.0232 (12)	0.0225 (12)	0.0330 (13)	−0.0118 (10)	−0.0019 (10)	0.0063 (10)
Br1A	0.02379 (12)	0.01843 (11)	0.01960 (11)	−0.00036 (9)	−0.00319 (9)	0.00021 (8)
Br2A	0.02581 (13)	0.02373 (13)	0.03068 (14)	−0.00786 (10)	−0.00669 (10)	0.00987 (10)
O1A	0.0182 (8)	0.0409 (11)	0.0200 (9)	−0.0068 (8)	−0.0022 (7)	0.0120 (8)
O2A	0.0272 (10)	0.0641 (15)	0.0212 (10)	0.0028 (10)	−0.0076 (8)	0.0062 (10)
C1A	0.0124 (9)	0.0250 (11)	0.0187 (11)	−0.0037 (8)	−0.0039 (8)	0.0067 (9)
C2A	0.0141 (10)	0.0168 (10)	0.0339 (13)	−0.0041 (8)	−0.0091 (9)	0.0083 (9)
C3A	0.0131 (10)	0.0183 (11)	0.0285 (12)	−0.0013 (8)	−0.0081 (9)	−0.0012 (9)
C4A	0.0136 (10)	0.0228 (11)	0.0175 (10)	−0.0032 (8)	−0.0035 (8)	−0.0003 (8)
C5A	0.0116 (9)	0.0193 (10)	0.0195 (11)	−0.0048 (8)	−0.0047 (8)	0.0027 (8)
C6A	0.0144 (9)	0.0192 (10)	0.0180 (10)	−0.0041 (8)	−0.0051 (8)	0.0000 (8)
C7A	0.0280 (12)	0.0197 (11)	0.0194 (11)	−0.0101 (10)	−0.0076 (9)	0.0039 (9)
C8A	0.0399 (15)	0.0364 (15)	0.0227 (13)	−0.0211 (13)	−0.0022 (11)	0.0086 (11)
C9A	0.0200 (12)	0.0241 (13)	0.0471 (17)	−0.0002 (10)	−0.0115 (11)	−0.0131 (12)
C10A	0.0176 (10)	0.0240 (12)	0.0267 (12)	−0.0091 (9)	−0.0050 (9)	0.0045 (9)

### 3,5-Bis(bromomethyl)phenyl acetate (2) Geometric parameters (Å, º)

**Table:**

Br1—C9	1.962 (2)	Br1A—C9A	1.960 (3)
Br2—C10	1.965 (3)	Br2A—C10A	1.979 (2)
O1—C7	1.362 (3)	O1A—C7A	1.353 (3)
O1—C1	1.407 (3)	O1A—C1A	1.403 (3)
O2—C7	1.196 (3)	O2A—C7A	1.184 (3)
C1—C2	1.379 (3)	C1A—C2A	1.379 (4)
C1—C6	1.383 (3)	C1A—C6A	1.380 (3)
C2—C3	1.392 (3)	C2A—C3A	1.390 (4)
C2—H2	0.9300	C2A—H2A	0.9300
C3—C4	1.392 (3)	C3A—C4A	1.389 (3)
C3—C9	1.492 (3)	C3A—C9A	1.498 (4)
C4—C5	1.389 (3)	C4A—C5A	1.392 (3)
C4—H4	0.9300	C4A—H4A	0.9300
C5—C6	1.391 (3)	C5A—C6A	1.391 (3)
C5—C10	1.495 (3)	C5A—C10A	1.488 (3)
C6—H6	0.9300	C6A—H6A	0.9300
C7—C8	1.492 (3)	C7A—C8A	1.494 (4)
C8—H8A	0.9600	C8A—H8A1	0.9600
C8—H8B	0.9600	C8A—H8A2	0.9600
C8—H8C	0.9600	C8A—H8A3	0.9600
C9—H9A	0.9700	C9A—H9A1	0.9700
C9—H9B	0.9700	C9A—H9A2	0.9700
C10—H10A	0.9700	C10A—H10C	0.9700
C10—H10B	0.9700	C10A—H10D	0.9700
			
C7—O1—C1	118.16 (18)	C7A—O1A—C1A	118.43 (19)
C2—C1—C6	122.2 (2)	C2A—C1A—C6A	121.8 (2)
C2—C1—O1	116.6 (2)	C2A—C1A—O1A	119.6 (2)
C6—C1—O1	121.1 (2)	C6A—C1A—O1A	118.3 (2)
C1—C2—C3	119.2 (2)	C1A—C2A—C3A	119.3 (2)
C1—C2—H2	120.4	C1A—C2A—H2A	120.3
C3—C2—H2	120.4	C3A—C2A—H2A	120.3
C4—C3—C2	119.3 (2)	C4A—C3A—C2A	119.4 (2)
C4—C3—C9	120.7 (2)	C4A—C3A—C9A	120.0 (2)
C2—C3—C9	120.0 (2)	C2A—C3A—C9A	120.5 (2)
C5—C4—C3	120.8 (2)	C3A—C4A—C5A	121.0 (2)
C5—C4—H4	119.6	C3A—C4A—H4A	119.5
C3—C4—H4	119.6	C5A—C4A—H4A	119.5
C4—C5—C6	119.9 (2)	C6A—C5A—C4A	119.2 (2)
C4—C5—C10	120.1 (2)	C6A—C5A—C10A	120.1 (2)
C6—C5—C10	120.0 (2)	C4A—C5A—C10A	120.7 (2)
C1—C6—C5	118.6 (2)	C1A—C6A—C5A	119.3 (2)
C1—C6—H6	120.7	C1A—C6A—H6A	120.3
C5—C6—H6	120.7	C5A—C6A—H6A	120.3
O2—C7—O1	123.3 (2)	O2A—C7A—O1A	122.4 (2)
O2—C7—C8	126.2 (2)	O2A—C7A—C8A	126.0 (2)
O1—C7—C8	110.5 (2)	O1A—C7A—C8A	111.6 (2)
C7—C8—H8A	109.5	C7A—C8A—H8A1	109.5
C7—C8—H8B	109.5	C7A—C8A—H8A2	109.5
H8A—C8—H8B	109.5	H8A1—C8A—H8A2	109.5
C7—C8—H8C	109.5	C7A—C8A—H8A3	109.5
H8A—C8—H8C	109.5	H8A1—C8A—H8A3	109.5
H8B—C8—H8C	109.5	H8A2—C8A—H8A3	109.5
C3—C9—Br1	111.76 (16)	C3A—C9A—Br1A	112.24 (17)
C3—C9—H9A	109.3	C3A—C9A—H9A1	109.2
Br1—C9—H9A	109.3	Br1A—C9A—H9A1	109.2
C3—C9—H9B	109.3	C3A—C9A—H9A2	109.2
Br1—C9—H9B	109.3	Br1A—C9A—H9A2	109.2
H9A—C9—H9B	107.9	H9A1—C9A—H9A2	107.9
C5—C10—Br2	111.29 (17)	C5A—C10A—Br2A	110.38 (16)
C5—C10—H10A	109.4	C5A—C10A—H10C	109.6
Br2—C10—H10A	109.4	Br2A—C10A—H10C	109.6
C5—C10—H10B	109.4	C5A—C10A—H10D	109.6
Br2—C10—H10B	109.4	Br2A—C10A—H10D	109.6
H10A—C10—H10B	108.0	H10C—C10A—H10D	108.1
			
C7—O1—C1—C2	−116.6 (2)	C7A—O1A—C1A—C2A	−81.9 (3)
C7—O1—C1—C6	66.7 (3)	C7A—O1A—C1A—C6A	104.9 (3)
C6—C1—C2—C3	−0.8 (4)	C6A—C1A—C2A—C3A	0.2 (3)
O1—C1—C2—C3	−177.5 (2)	O1A—C1A—C2A—C3A	−172.7 (2)
C1—C2—C3—C4	0.2 (3)	C1A—C2A—C3A—C4A	−0.4 (3)
C1—C2—C3—C9	−178.3 (2)	C1A—C2A—C3A—C9A	175.3 (2)
C2—C3—C4—C5	0.4 (3)	C2A—C3A—C4A—C5A	0.8 (3)
C9—C3—C4—C5	178.8 (2)	C9A—C3A—C4A—C5A	−175.0 (2)
C3—C4—C5—C6	−0.3 (3)	C3A—C4A—C5A—C6A	−0.9 (3)
C3—C4—C5—C10	177.8 (2)	C3A—C4A—C5A—C10A	178.1 (2)
C2—C1—C6—C5	0.9 (4)	C2A—C1A—C6A—C5A	−0.3 (3)
O1—C1—C6—C5	177.4 (2)	O1A—C1A—C6A—C5A	172.73 (19)
C4—C5—C6—C1	−0.3 (3)	C4A—C5A—C6A—C1A	0.6 (3)
C10—C5—C6—C1	−178.5 (2)	C10A—C5A—C6A—C1A	−178.4 (2)
C1—O1—C7—O2	−4.0 (3)	C1A—O1A—C7A—O2A	−5.1 (4)
C1—O1—C7—C8	174.9 (2)	C1A—O1A—C7A—C8A	175.6 (2)
C4—C3—C9—Br1	70.6 (2)	C4A—C3A—C9A—Br1A	−95.8 (3)
C2—C3—C9—Br1	−111.0 (2)	C2A—C3A—C9A—Br1A	88.5 (3)
C4—C5—C10—Br2	80.7 (2)	C6A—C5A—C10A—Br2A	99.1 (2)
C6—C5—C10—Br2	−101.1 (2)	C4A—C5A—C10A—Br2A	−79.9 (2)

### 3,5-Bis(bromomethyl)phenyl acetate (2) Hydrogen-bond geometry (Å, º)

**Table:**

*D*—H···*A*	*D*—H	H···*A*	*D*···*A*	*D*—H···*A*
C10*A*—H10*D*···O2*A*^i^	0.97	2.28	3.236 (3)	168
C10*A*—H10*C*···Br1*A*^i^	0.97	2.89	3.836 (3)	164
C8*A*—H8*A*3···O2	0.96	2.58	3.521 (4)	168
C10—H10*B*···Br2*A*^i^	0.97	3.01	3.757 (3)	135
C10—H10*A*···O2^ii^	0.97	2.58	3.449 (3)	150
C9—H9*B*···Br2^iii^	0.97	2.95	3.854 (3)	156
C9—H9*A*···O2^iii^	0.97	2.45	3.334 (3)	151

Symmetry codes: (i) −*x*+1, −*y*, −*z*+1; (ii) *x*−1, *y*, *z*; (iii) −*x*+1, −*y*+1, −*z*.

### 5-Hydroxybenzene-1,3-dicarbaldehyde (3) Crystal data

**Table:**

C_8_H_6_O_3_	*F*(000) = 312
*M**_r_* = 150.13	*D*_x_ = 1.485 Mg m^−^^3^
Monoclinic, *P*2_1_/*n*	Mo *K*α radiation, λ = 0.71073 Å
*a* = 3.7345 (1) Å	Cell parameters from 6158 reflections
*b* = 11.9549 (4) Å	θ = 2.7–30.5°
*c* = 15.0846 (5) Å	µ = 0.12 mm^−^^1^
β = 94.212 (2)°	*T* = 153 K
*V* = 671.64 (4) Å^3^	Rod, colourless
*Z* = 4	0.42 × 0.28 × 0.19 mm

### 5-Hydroxybenzene-1,3-dicarbaldehyde (3) Data collection

**Table:**

Bruker Kappa APEXII CCD area detector diffractometer	*R*_int_ = 0.058
φ and ω scans	θ_max_ = 29.4°, θ_min_ = 2.7°
11533 measured reflections	*h* = −5→4
1819 independent reflections	*k* = −16→16
1519 reflections with *I* > 2σ(*I*)	*l* = −20→20

### 5-Hydroxybenzene-1,3-dicarbaldehyde (3) Refinement

**Table:**

Refinement on *F*^2^	0 restraints
Least-squares matrix: full	Hydrogen site location: mixed
*R*[*F*^2^ > 2σ(*F*^2^)] = 0.047	H atoms treated by a mixture of independent and constrained refinement
*wR*(*F*^2^) = 0.131	*w* = 1/[σ^2^(*F*_o_^2^) + (0.0692*P*)^2^ + 0.2868*P*] where *P* = (*F*_o_^2^ + 2*F*_c_^2^)/3
*S* = 1.06	(Δ/σ)_max_ < 0.001
1819 reflections	Δρ_max_ = 0.33 e Å^−^^3^
104 parameters	Δρ_min_ = −0.28 e Å^−^^3^

### 5-Hydroxybenzene-1,3-dicarbaldehyde (3) Special details

**Table:**

Geometry. All esds (except the esd in the dihedral angle between two l.s. planes) are estimated using the full covariance matrix. The cell esds are taken into account individually in the estimation of esds in distances, angles and torsion angles; correlations between esds in cell parameters are only used when they are defined by crystal symmetry. An approximate (isotropic) treatment of cell esds is used for estimating esds involving l.s. planes.

### 5-Hydroxybenzene-1,3-dicarbaldehyde (3) Fractional atomic coordinates and isotropic or equivalent isotropic displacement parameters (Å^2^)

**Table:**

	*x*	*y*	*z*	*U*_iso_*/*U*_eq_	
O1	0.6355 (3)	0.48950 (8)	0.38804 (7)	0.0332 (3)	
O2	1.0631 (3)	0.10838 (8)	0.62211 (7)	0.0336 (3)	
O3	0.2117 (3)	0.11521 (8)	0.23777 (6)	0.0291 (3)	
C1	0.6468 (4)	0.37799 (10)	0.40378 (8)	0.0214 (3)	
C2	0.8207 (3)	0.34507 (10)	0.48515 (8)	0.0207 (3)	
H2	0.9189	0.4000	0.5254	0.025*	
C3	0.8496 (3)	0.23282 (10)	0.50697 (7)	0.0197 (3)	
C4	0.7080 (4)	0.15111 (10)	0.44830 (8)	0.0214 (3)	
H4	0.7294	0.0740	0.4631	0.026*	
C5	0.5354 (3)	0.18440 (10)	0.36798 (8)	0.0206 (3)	
C6	0.5036 (3)	0.29757 (10)	0.34512 (8)	0.0204 (3)	
H6	0.3850	0.3191	0.2899	0.024*	
C7	1.0363 (4)	0.20285 (11)	0.59351 (8)	0.0235 (3)	
H7	1.1419	0.2615	0.6290	0.028*	
C8	0.3862 (4)	0.09684 (11)	0.30752 (9)	0.0253 (3)	
H8	0.4289	0.0210	0.3240	0.030*	
H1	0.519 (7)	0.5065 (19)	0.3394 (16)	0.056 (7)*	

### 5-Hydroxybenzene-1,3-dicarbaldehyde (3) Atomic displacement parameters (Å^2^)

**Table:**

	*U*^11^	*U*^22^	*U*^33^	*U*^12^	*U*^13^	*U*^23^
O1	0.0477 (7)	0.0190 (5)	0.0297 (5)	−0.0017 (4)	−0.0184 (5)	0.0031 (4)
O2	0.0445 (7)	0.0281 (5)	0.0269 (5)	0.0034 (4)	−0.0066 (4)	0.0052 (4)
O3	0.0329 (6)	0.0298 (5)	0.0233 (5)	−0.0024 (4)	−0.0071 (4)	−0.0047 (4)
C1	0.0228 (7)	0.0204 (6)	0.0203 (5)	−0.0004 (4)	−0.0036 (4)	0.0005 (4)
C2	0.0214 (7)	0.0217 (6)	0.0183 (5)	−0.0005 (4)	−0.0034 (4)	−0.0010 (4)
C3	0.0184 (6)	0.0228 (6)	0.0175 (5)	0.0005 (4)	−0.0012 (4)	0.0005 (4)
C4	0.0227 (7)	0.0206 (5)	0.0204 (5)	0.0000 (4)	−0.0011 (4)	0.0002 (4)
C5	0.0193 (6)	0.0234 (6)	0.0187 (5)	−0.0008 (4)	−0.0011 (4)	−0.0025 (4)
C6	0.0194 (6)	0.0236 (6)	0.0175 (5)	−0.0006 (4)	−0.0023 (4)	−0.0003 (4)
C7	0.0248 (7)	0.0257 (6)	0.0195 (5)	0.0022 (5)	−0.0027 (4)	0.0006 (4)
C8	0.0267 (7)	0.0248 (6)	0.0236 (6)	−0.0025 (5)	−0.0026 (5)	−0.0026 (5)

### 5-Hydroxybenzene-1,3-dicarbaldehyde (3) Geometric parameters (Å, º)

**Table:**

O1—C1	1.3541 (15)	C3—C7	1.4781 (16)
O1—H1	0.85 (2)	C4—C5	1.3882 (16)
O2—C7	1.2105 (16)	C4—H4	0.9500
O3—C8	1.2163 (16)	C5—C6	1.3991 (17)
C1—C6	1.3870 (16)	C5—C8	1.4709 (17)
C1—C2	1.4022 (16)	C6—H6	0.9500
C2—C3	1.3840 (17)	C7—H7	0.9500
C2—H2	0.9500	C8—H8	0.9500
C3—C4	1.3958 (16)		
			
C1—O1—H1	113.2 (16)	C4—C5—C6	121.19 (11)
O1—C1—C6	124.40 (11)	C4—C5—C8	117.88 (11)
O1—C1—C2	115.87 (11)	C6—C5—C8	120.92 (11)
C6—C1—C2	119.73 (11)	C1—C6—C5	119.43 (11)
C3—C2—C1	120.23 (11)	C1—C6—H6	120.3
C3—C2—H2	119.9	C5—C6—H6	120.3
C1—C2—H2	119.9	O2—C7—C3	124.21 (12)
C2—C3—C4	120.56 (11)	O2—C7—H7	117.9
C2—C3—C7	117.95 (11)	C3—C7—H7	117.9
C4—C3—C7	121.49 (11)	O3—C8—C5	124.23 (12)
C5—C4—C3	118.86 (11)	O3—C8—H8	117.9
C5—C4—H4	120.6	C5—C8—H8	117.9
C3—C4—H4	120.6		
			
O1—C1—C2—C3	−179.31 (12)	O1—C1—C6—C5	179.13 (13)
C6—C1—C2—C3	0.0 (2)	C2—C1—C6—C5	−0.1 (2)
C1—C2—C3—C4	0.3 (2)	C4—C5—C6—C1	−0.1 (2)
C1—C2—C3—C7	179.88 (12)	C8—C5—C6—C1	179.87 (12)
C2—C3—C4—C5	−0.5 (2)	C2—C3—C7—O2	176.10 (14)
C7—C3—C4—C5	179.96 (12)	C4—C3—C7—O2	−4.3 (2)
C3—C4—C5—C6	0.3 (2)	C4—C5—C8—O3	175.61 (14)
C3—C4—C5—C8	−179.58 (12)	C6—C5—C8—O3	−4.3 (2)

### 5-Hydroxybenzene-1,3-dicarbaldehyde (3) Hydrogen-bond geometry (Å, º)

**Table:**

*D*—H···*A*	*D*—H	H···*A*	*D*···*A*	*D*—H···*A*
C2—H2···O1^i^	0.95	2.43	3.3354 (16)	160
C8—H8···O2^ii^	0.95	2.58	3.1973 (18)	123
O1—H1···O3^iii^	0.85 (2)	1.91 (2)	2.6795 (13)	150 (2)

Symmetry codes: (i) −*x*+2, −*y*+1, −*z*+1; (ii) −*x*+1, −*y*, −*z*+1; (iii) −*x*+1/2, *y*+1/2, −*z*+1/2.
